# FOXC1 Negatively Regulates DKK1 Expression to Promote Gastric Cancer Cell Proliferation Through Activation of Wnt Signaling Pathway

**DOI:** 10.3389/fcell.2021.662624

**Published:** 2021-04-27

**Authors:** Jiang Jiang, Jianfang Li, Weiwu Yao, Wenfang Wang, Bowen Shi, Fei Yuan, Jingyan Dong, Huan Zhang

**Affiliations:** ^1^Department of Radiology, Ruijin Hospital, Shanghai Jiao Tong University School of Medicine, Shanghai, China; ^2^Department of General Surgery, Shanghai Key Laboratory of Gastric Neoplasms, Shanghai Institute of Digestive Surgery, Ruijin Hospital, Shanghai Jiao Tong University School of Medicine, Shanghai, China; ^3^Department of Radiology, Tongren Hospital, Shanghai Jiao Tong University School of Medicine, Shanghai, China; ^4^Department of Pathology, Ruijin Hospital, Shanghai Jiao Tong University School of Medicine, Shanghai, China; ^5^Department of Ocular Fundus Diseases, Shanxi Eye Hospital, Shanxi, China

**Keywords:** gastric cancer, FOXC1, DKK1, c-MYC, proliferation

## Abstract

Gastric cancer (GC), characterized by uncontrolled growth, is a common malignant tumor of the digestive system. The Wnt signaling pathway plays an important role in the tumorigenesis and proliferation of GC. Many studies on this signaling pathway have focused on its intracellular regulatory mechanism, whereas little attention has been given to extracellular regulatory factors. Dickkopf-1 (Dkk1) is a secretory glycoprotein, and it can bind inhibit activation of the Wnt pathway. However, the regulation and mechanism of DKK1 in the proliferation of GC remain unclear. FOXC1 plays an important role in organ development and tumor growth, but its role in GC tumor growth remains unknown. In this study, we found that the FOXC1 is highly expressed in patients with GC and high expression of FOXC1 correlates to poor prognosis. In addition, we found that the Wnt signaling pathway in GC cells with high FOXC1 expression was strongly activated. FOXC1 negatively regulates DKK1 expression by binding to its promoter region, thereby promoting the activation of Wnt pathway. FOXC1 can also form a complex with unphosphorylated β-catenin protein in the cytoplasm and then dissociates from β-catenin in the nucleus, thereby promoting the entry of β-catenin into the nucleus and regulating expression of c-MYC, which promotes the proliferation of GC cells. Our study not only reveals the function and mechanism of FOXC1 in GC, but also provides a potential target for clinic GC treatment.

## Introduction

Gastric cancer (GC) is one of the most common malignant tumors in the digestive system, especially in East Asia. In 2013, approximately 984,000 people were diagnosed with GC, and nearly 841,000 died of GC (Fitzmaurice et al., 2015). Due to the lack of specific tumor biomarkers, many tumors were at an advanced stage when diagnosed and patients have lost the opportunity for radical surgical treatment, for those patients, their 5-year survival rate is less than 10% (Shimada et al., 2014). Thus, identifying the underlying mechanisms and specific biomarkers is a pivotal project to treat GC.

Forkhead box (FOX) proteins are characterized by a winged helix DNA-binding domain (Erickson, 2001). These proteins have been shown to regulate diverse biological processes, including development, differentiation, proliferation, apoptosis, migration, invasion, and tumorigenesis (Jin et al., 2014). Forkhead box C1 (FOXC1), a member of the FOX protein family, is a key regulator of the development of the anterior chamber angle, and its abnormal expression correlates highly with the incidence of congenital glaucoma (Chen et al., 2016; Golson and Kaestner, 2016; Deng et al., 2017; Ramezani et al., 2019). Recent studies have shown that FOXC1 dysregulation is strongly related to the occurrence and development of breast cancer, colon cancer, cervical cancer, Hodgkin’s lymphoma, and prostate cancer (Ito et al., 2014; Jin et al., 2014; Hopkins et al., 2016; Gilding and Somervaille, 2019; Hsu et al., 2019). In 2014, Yuan et al. reported that FOXC1 was associated with tumor size, the number of lymph node metastases, and prognosis, which indicated that FOXC1 might play an important role in GC growth and metastasis (Xu et al., 2014). Zhong et al. reported that LINC00242 interacts with miR-141 and positively regulates FOXC1 to contribute to HGC27 cell viability, migration, and invasion (Zhong et al., 2020). Moreover, previous studies have indicated that FOXC1 promotes tumor proliferation through the PI3K-AKT, NF-KB and Wnt signaling pathways (Huang et al., 2017; Liu et al., 2018; Yu et al., 2018). However, its specific role in proliferation of GC remains to be studied.

Wnt signaling pathway play an important role in embryonic development, cell migration, and organogenesis (Chae and Bothwell, 2018). Its aberrant activation could contribute GC growth and metastasis (Zhan et al., 2017). In the classical Wnt signaling pathway, Wnt protein binds to cell surface receptors, including Frizzled (Fz) and low-density lipoprotein receptor-related protein 5/6 (LRP5/6), thereby transducing extracellular signal stimuli into cells (Reya and Clevers, 2005). Wnt proteins formed a complex with Fzd and LRP5/6, Which led to the level of phosphorylation β-catenin reduced and excessive accumulation of β-catenin in the cytoplasm; it then enters into the nucleus and forms a transcriptional complex with TCF or LEF, activating expression of target genes, such as c-MYC and cyclin D1 (Clevers and Nusse, 2012). Among them, the transcription factor c-Myc is one of the most widely investigated cancer-causing genes, being implicated in the formation, maintenance, and progression of several different cancer types (Duffy et al., 2021). Previous study has indicated that c-Myc is well-characterized β-catenin/TCF target gene (Sansom et al., 2007). And overexpression of MYC can rescue the growth-suppressive effects of FZD7 knockdown in gastric cancer cells (Flanagan et al., 2019). Previous study reported that FOXC1 knockdown could inhibit the expression of MYC, however, the precise role and underlying signaling cascades in GC proliferation remain unclear.

Dickkopf-1 (Dkk1) is a secretory antagonist, which binds to the Wnt coreceptor LRP5/6 to desensitize cells to canonical Wnt ligands (Chae and Bothwell, 2018). DKK-1 is reported to be over expressed in GC patients and recently, it was reported to play different roles in the tumor growth due to different tumor environment (Gomceli et al., 2012; Zhu et al., 2021). In 2018, Hong et al. reported that high DKK1 expression is a crucial prognostic factor for predicting tumor recurrence and survival in patients with resected advanced GC, which indicated DKK1 contributed GC recurrence (Hong et al., 2018). Meanwhile, in terms of tumor growth, DKK-1 inhibits the activation of Wnt signaling to influence GC growth by suppressing cancer stem cells (Wang et al., 2013). Therefore, it is particularly important to clearly explain the specific mechanism of DKK1 in the proliferation of GC. However, there are few studies to explore transcription factors that regulate DKK1 in GC.

In this study, we will explore the specific mechanisms that FOXC1 promoted proliferation GC by inhibiting DKK1 expression.

## Materials and Methods

### Patients and Human Tissue Information

Tumor tissue samples were obtained from Ruijin Hospital, Shanghai Jiao Tong University. Patients underwent curative GC resection between 2018 and 2019, and samples from these patients were used for immunohistochemistry (IHC). The Ethics Committee of Ruijin Hospital, Shanghai Jiao Tong University approved this project. All samples were anonymously coded in accordance with local ethical guidelines (as stipulated by the Declaration of Helsinki), and written informed consent was obtained.

### Cell Culture and Reagents

Normal gastric cell (GES-1) and GC cell lines (AGS, SGC-7901, MGC-803, MKN-45, HGC-27) were stored at Shanghai Institute of Digestive Surgery, Ruijin Hospital. Cell lines were cultured in RPMI-1640 medium or Dulbecco’s modified Eagle’s medium (DMEM) with 10% fetal bovine serum (Gibco, United States) and 5 μg/ml penicillin-streptomycin in a humidified incubator at 37°C with 5% CO_2_.

### Quantitative Real-Time PCR

Total cellular RNA was isolated from GC cells according to the EZB RNA reagent kit protocol, and 200 ng of total RNA was reverse transcribed to cDNA using a PrimeScript RT Master Mix Kit (Takara Bio). mRNA levels were measured by quantitative real-time PCR using SYBR Premix Ex Taq, and human GAPDH was used as an internal control.

### Western Blot Analysis of Gene Expression

Western blotting was performed as described previously (Jiang et al., 2018). Antibodies against FOXC1, DKK1, c-MYC, and β-catenin were purchased from Abcam, and the secondary antibody (anti-rabbit IgG or anti-mouse IgG) was purchased from Proteintech. An anti-GAPDH antibody was used as an internal control. Detail information could be obtained in the Supplementary Material 2.

### Expression Vectors and Gene Transfection

Plasmid transfection process was performed using Lipofectamine 2000 Reagent (Life Technology, Thermo Fisher Scientific, DE, United States). Full-length FOXC1 cDNAs were cloned into the pLVXyu-2Flag-3C-2 vector for FOXC1 expression. The c-MYC plasmid and DKK plasmid were gifts from Professor Guohong Hu at The Key Laboratory of Stem Cell Biology, Institute of Health Sciences, Shanghai Institutes for Biological Sciences. Stably transfected GC cells were established using puromycin selection after transfection with the expression vector or control plasmid. CRISPR/Cas9 vector plasmid was obtained from the Molecular and Cell Biology Laboratory, Fudan University. The sgRNA target sequence for FOXC1 was 5′-GGGTGCGAGTACACGCTCAT-3′, and CRISPR/Cas9 vector plasmid was used as a control. Stably transfected GC cells for 24 h were established using puromycin selection for 2 weeks after transfection. Knockout effiency were validated by Western blot with FOXC1 antibody.

### Luciferase Assay

The pGL3-Basic Luciferase Reporter vector was a gift from Fudan University Shanghai Cancer Center. The 2.1-kb DKK1 and c-MYC promoter was cloned into the pGL3-Basic Luciferase Reporter vector. Activity of the DKK1 and c-MYC promoters was normalized by co-transfection with the Renilla luciferase reporter plasmid, which was a gift from Fudan University. Firefly and Renilla luciferase activities were measured at 48 h after transfection using a Dual-Luciferase Reporter Assay System (Promega).

### TOP-Flash/FOP-Flash Reporter Assay

A TOP-flash/FOP-flash-dependent luciferase reporter assay was used to evaluate Wnt/LEF/TCF- activity. TOP-flash/FOP flash and Renilla plasmids to demonstrate transfection efficiency were incubated for 24 h. Relative luciferase activity was determined using a dual-luciferase assay (Promega). Transfection efficiency was normalized according to Renilla luciferase activity. The cells were lysed at 16 h after transfection, and luciferase activities were determined.

### Chromatin Immunoprecipitation Assay

GC cells (2^∗^10^^7^) were used for chromatin immunoprecipitation assays, and an anti-FOXC1 antibody was used to pull down DKK1 and c-MYC promoter-protein complexes. This assay was performed using CST Chromatin Immunoprecipitation Kit (CST9002) according to the manufacturer’s instructions. The DNA samples were analyzed by PCR for potential binding sites. The PCR products were separated and visualized on a 2.5% agarose gel. The primers used for PCR are listed in Table 1.

**TABLE 1:** Quantitative real time polymerase chain reaction assay (Q-PCR) primer.

**Gene name**	**Sequence detail information**
GAPDH-Forward	GCACCGTCAAGGCTGAGAAC
GAPDH-Reverse	TGGTGAAGACGCCAGTGGA
FOXC1 -Forward	AACAGCATCCGCCACAACCTC
FOXC1-Reverse	TCCTTCTCCTCCTTGTCCTTCAC
YAP-Forward	TGTCCCAGATGA ACGTCACAGC
YAP-Reverse	TGGTGGCTGTTT CACTGGAGCA
TAZ -Forward	CACCGTGTCCAATCACCAGTC
TAZ -Reverse	TCCAACGCATCAACTTCAGGT
β-catenin -Forward	CATCTACACAGTTTGATGCTGCT
β-catenin - Reverse	GCAGTTTTGTCAGTTCAGGGA
AXIN1 -Forward	GGTTTCCCCTTGGACCTCG
AXIN1 -Reverse	CCGTCGAAGTCTCACCTTTAATG
NOTCH1 -Forward	GAGGCGTGGCAGACTATGC
NOTCH1 -Reverse	CTTGTACTCCGTCAGCGTGA
BIRC5 -Forward	AGGACCACCGCATCTCTACAT
BIRC5 -Reverse	AAGTCTGGCTCGTTCTCAGTG
CCND1-Forward	GCTGCGAAGTGGAAACCATC
CCND1-Reverse	CCTCCTTCTGCACACATTTGAA
c-MYC -Forward	GTCAAGAGGCGAACACACAAC
c-MYC -Reverse	TTGGACGGACAGGATGTATGC
SPP1 -Forward	GAAGTTTCGCAGACCTGACAT
SPP1 -Reverse	GTATGCACCATTCAACTCCTCG
GPC3 -Forward	ATTGGCAAGTTATGTGCCCAT
GPC3 -Reverse	TTCGGCTGGATAAGGTTTCTTC
BAX -Forward	CCCGAGAGGTCTTTTTCCGAG
BAX -Reverse	CCAGCCCATGATGGTTCTGAT
PUMA-Forward	GCCAGATTTGTGAGACAAGAGG
PUMA-Reverse	CAGGCACCTAATTGGGCTC
DKK1 -Forward	CTCGGTTCTCAATTCCAACG
DKK1 -Reverse	GCACTCCTCGTCCTCTG

### Immunofluorescence (IF) Staining and Confocal Microscopy

AGS and MKN-45 cells were seeded onto coverslips before they reached 80% confluence. The cells were fixed with 4% paraformaldehyde for 15 min, and the samples were kept in phosphate-buffered saline (PBS) containing 0.1% Triton X-100 (PBS-T), quenched with 50 mM NH4Cl in PBS-T, and blocked with 1% BSA in PBS-T. Immunostaining was performed using the appropriate primary and secondary antibodies, and images were acquired using a confocal microscope (Zeiss, LSM510).

### Apoptosis Analysis and Cell Cycle Analysis by Flow Cytometry

Apoptosis was evaluated by flow cytometry. Briefly, cells were stained with Annexin V-FITC conjugate and propidium iodide solution. A FACSCalibur system (BD Biosciences, United States) was used to analyze apoptosis.

The cells were fixed in 75% ethanol and stained with PI/RNase Staining Buffer (BD Biosciences). The cell cycle was analyzed by flow cytometry using the FACSCalibur system (BD Biosciences, United States).

### Cell Proliferation and Colony Formation Assay

GC cell proliferation was evaluated by the CCK-8 assay. For the colony formation assay, cells were seeded into six-well culture plates at a density of 1,000–2,000 cells/well and grown for 10–14 days. The cells were fixed with methanol and stained with 0.1% crystal violet.

### Mouse Models of GC Tumorigenesis

Ten nude mice were sorted into two groups, and GC cells (5 × 10^6^ per mouse) were subcutaneously injected. The mice were sacrificed 5 weeks later. The tumors were removed and weighed, processed and embedded in paraffin for further study.

### Statistical Analysis

All experiments that were presented in this work were repeated more than three times. Data was presented as mean ± standard error of the mean. For statistical analysis, two-tailed independent Student’s t-test was applied to a demonstration of homogeneity of variance with the F test or one-way or two-way ANOVA for more than two groups. Statistical significance was set as *P* < 0.05.

## Results

### FOXC1 Expression Is Upregulated in Human Gastric Cancer Tissues and Cell Lines

**FIGURE 1:**
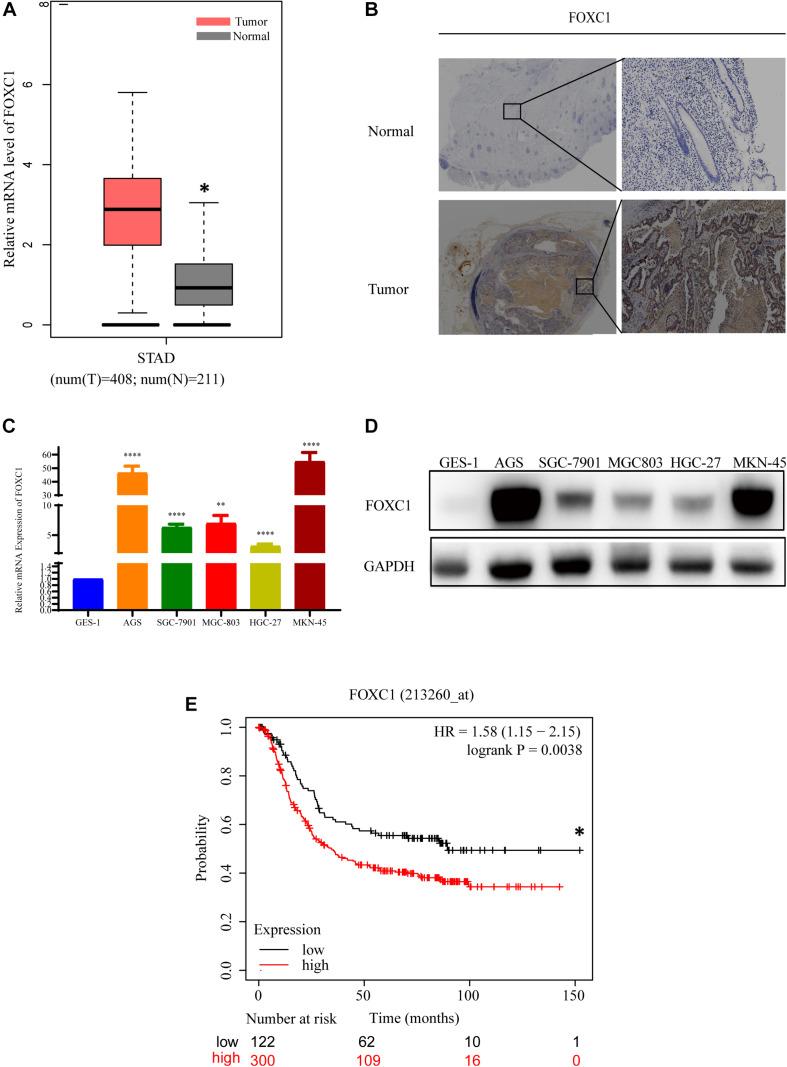
FOXC1 expression is upregulated in human gastric cancer tissues and cell lines. **(A)** Comparison of relative mRNA levels of FOXC1 in gastric cancer and normal tissues. The data were downloaded from GEPIA (http://gepia.cancer-pku.cn/detail.php?gene = FOXC1). **(B)** IHC staining was applied to detect FOXC1 protein levels in gastric cancer and normal tissue. FOXC1 expression level was higher in 85.7% (18/21) of the GC tissues and representative images show increased FOXC1 expression in GC tissue (below panel) compared with adjacent normal gastric tissues (upper panel). **(C,D)** mRNA and protein levels of FOXC1 in the gastric cancer cell lines AGS, SGC-7901, MGC-803, MKN-45, HGC-27, and the GES-1 cell line. The expression levels of FOXC1 were normalized to GAPDH. (^∗^*P* < 0.05, ^∗∗^*P* < 0.01, ^****^P < 0.001). **(E)** Kaplan-Meier curves for the survival time of patients with gastric cancer. The data was obtained from Kaplan-Meier Plotter and affy ID of FOXC1(213260_at) dataset used for the analysis (http://kmplot.com/analysis/index.php?p = service). High and low expression groups are 300 and 122, respectively. The median survival time for high and low expression groups are 89.43 and 34.3 months respectively (*p* = 0.0038). Log-rank tests were used to determine statistical significance (*P < 0.05).

To investigate the role of FOXC1 in human gastric cancer, the mRNA level data of FOXC1 in GC and adjacent normal tissues were obtained from GEPIA and analyzed. And the result indicated that the expression level of FOXC1 was increased in GC tissues compared with normal tissues (Figure 1A). To confirm the expression level of FOXC1 in GC, IHC staining was applied to detect the protein level of FOXC1. As shown in Figure 1B, compared to normal tissues, the protein level of FOXC1 was higher in 85.7% (18/21) of the GC tissues. qPCR and western blot were applied to evaluate mRNA and protein levels of FOXC1 in non-malignant (GES-1) and gastric cancer cell lines (AGS, SGC-7901, MGC-803, MKN-45, HGC-27). The results demonstrated that both mRNA and protein levels were significantly upregulated in GC cell lines (Figures 1C,D). The data was obtained from Kaplan-Meier Plotter and affy ID of FOXC1(213260_at) dataset used for the analysis^1^. A total of 422 patients were concluded in our analysis and cut-off value for the high and low expression is 141. And number of patients in high and low expression groups are 300 and 122, respectively. The median survival time for high and low expression groups are 89.43 and 34.3 months respectively (*p* = 0.0038). In general, high expression of FOXC1 indicated a poor prognosis in GC (Figure 1E). These results indicate that FOXC1 might be an important oncogene in GC.

### FOXC1 Contributes to Gastric Cancer Growth *in vitro* and *in vivo*

**FIGURE 2:**
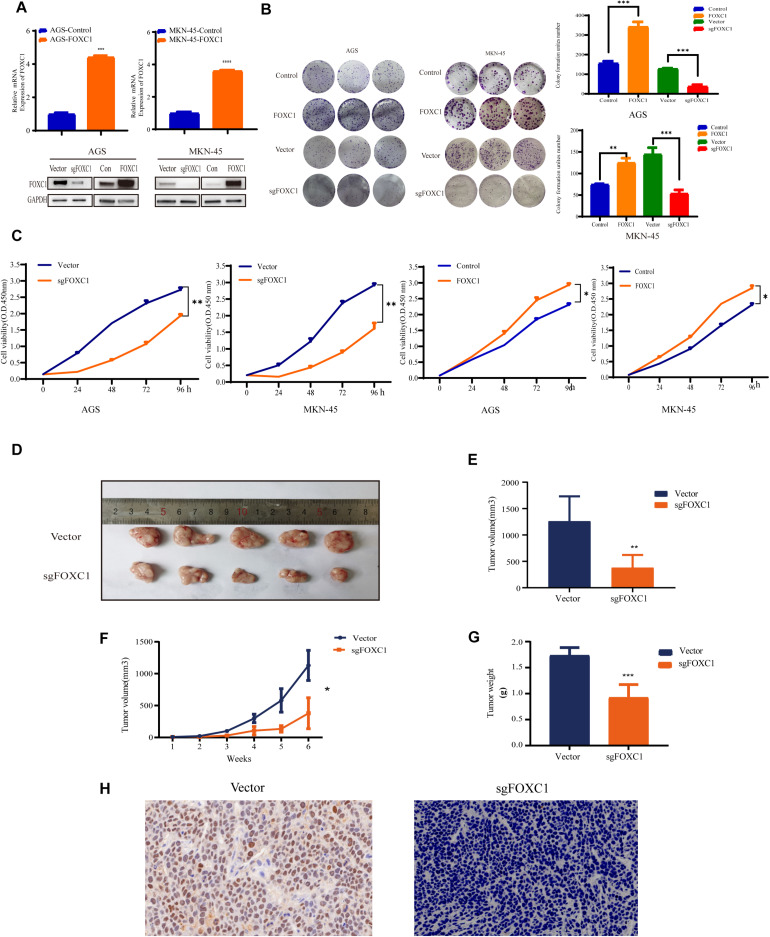
FOXC1 contributes to gastric cancer growth *in vitro* and *in vivo*. **(A)** Verification of overexpression in AGS and MKN-45 cells using qPCR. FOXC1 knockout and overexpress efficiency was confirmed using western blotting, and GAPDH was used as an internal control. **(B,C)** The impact of FOXC1 expression *in vitro* on GC cell proliferation, as assessed using colony formation assays **(B)**. **(C)** CCK-8 proliferation assays in FOXC1 KO and overexpress AGS and MKN-45 cells, respectively. All CCK-8 assays were performed three times and showed the same trend and representative results were shown in panel **(C)**. **(D–G)** MKN-45-Vector and MKN-45-sgFOXC1 cells were injected subcutaneously into right forelimb five nude mice, respectively (5 × 10^6^ cells for each mouse). Tumor size **(D)**, tumor volume **(E)**, tumor growth curves **(F)**, tumor weights **(G)** and IHC **(H)** are shown (^∗^*P* < 0.05, **P < 0.01, ***P < 0.001, ****P < 0.0001).

To investigate the role of FOXC1 in the tumorigenesis and progression of GC, we applied CRISPR–Cas9-mediated gene-editing system to knock out FOXC1 expression in AGS and MKN-45 cells. Meanwhile, FOXC1 sequence was cloned into pCDNA plasmids overexpressed FOXC1 in AGS and MKN-45 cells. The efficiencies were confirmed by western blot and qPCR assays (Figure 2A). We observed that FOXC1 knockout (KO) significantly suppressed colony formation and cell proliferation, while FOXC1 overexpression dramatically enhanced cell viability and proliferation (*p* < 0.05) (Figures 2B,C). To study the oncogenic functions of FOXC1 in GC, we checked the effect of FOXC1 knockout on tumor growth and found that FOXC1 KO significantly decreased the tumor growth of MKN-45 cells (*p* < 0.05) (Figures 2D–G). FOXC1 expression level in xenografts was determined by IHC (Figure 2H).

### Altered Expression of FOXC1 Affects the Cell Cycle in GC

**FIGURE 3:**
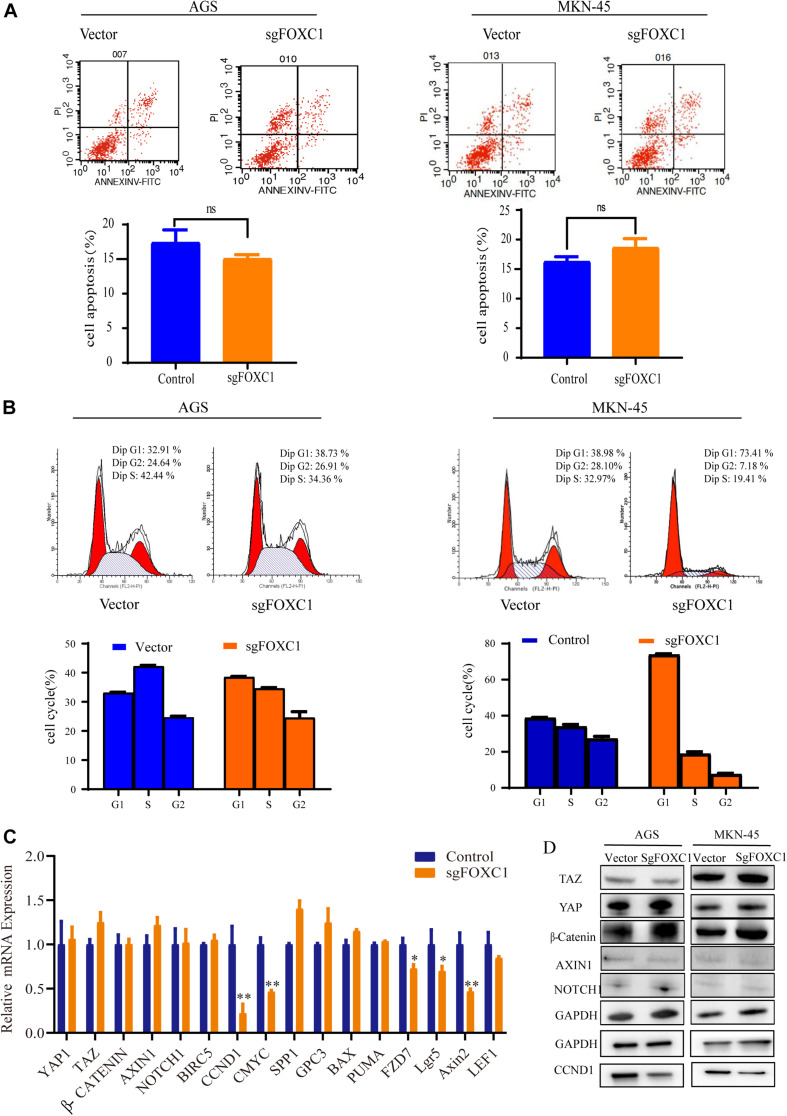
Altered expression of FOXC1 affects the cell cycle in GC. **(A)** Annexin-V & Dead Cell (7-AAD) flow cytometry analysis to evaluate cell apoptosis. In the right-down panel in each plot was considered as early meaningful apoptosis. Representative figure of the three replicates of each proportion of early apoptosis was shown in panel **(A)**. The results flow cytometry assay on cell apoptosis in FOXC1 KO cell lines (AGS cells and MKN-45 cells) had no significant influence on cell apoptosis. Values are presented as the mean ± standard deviation of three independent experiments (*P* > 0.05). **(B)** The results flow cytometry assay on cell cycle in FOXC1 KO cells lines (AGS cells and MKN-45 cells) showed that FOXC1 had a significant role in cell cycle regulation. Representative figure of the three replicates of each proportion of cell cycle was shown in panel **(B)**. Values are presented as the mean ± standard deviation of three independent experiments (^∗^*P* < 0.05, **P < 0.01). **(C,D)** Expression of core components of the Hippo, Wnt and Notch signaling pathways was determined by qPCR in FOXC1 KO AGS cells **(C)** and western blot **(D)** analyses in FOXC1 KO AGS and MKN-45 cells. ns, no significant difference and *P* > 0.05.

Apoptosis and cell cycle are two main factors that affect tumor growth. To investigate the role of FOXC1, we applied flow cytometry for verification. The results demonstrated that FOXC1 KO in GC cells didn’t affect apoptosis (Figure 3A), but decreased cell cycle progression (*p* < 0.05) (Figure 3B), indicating FOXC1 regulates the proliferation of GC cells through cell cycle. To search for downstream effectors of FOXC1-induced GC proliferation, we examined the expression levels of several signaling molecules (e.g., Wnt/β-catenin, Notch, and Hippo) implicated in the regulation of cell proliferation during gastric carcinogenesis. qPCR and western blotting analyses revealed unchanged expression of the core components of those signaling pathways with FOXC1 KO (Figures 3C,D). To better understand the role of FOXC1 in the cell cycle of GC, we further measured mRNA levels of other target genes, such as baculoviral IAP repeat-containing protein 5 (BIRC5), cyclin D1 (CCND1), c-MYC, secreted phosphoprotein 1 (SPP1), and glypican 3 (GPC3) BAX and PUMA and found that CCND1 and c-MYC gene expression was downregulated in FOXC1 KO AGS cells (Figure 3C), demonstrating that FOXC1 promotes cell cycle via regulation of cyclin D1 and c-MYC.

### FOXC1 Expression Enhances c-MYC Expression to Promote Gastric Cancer Cell Proliferation

**FIGURE 4:**
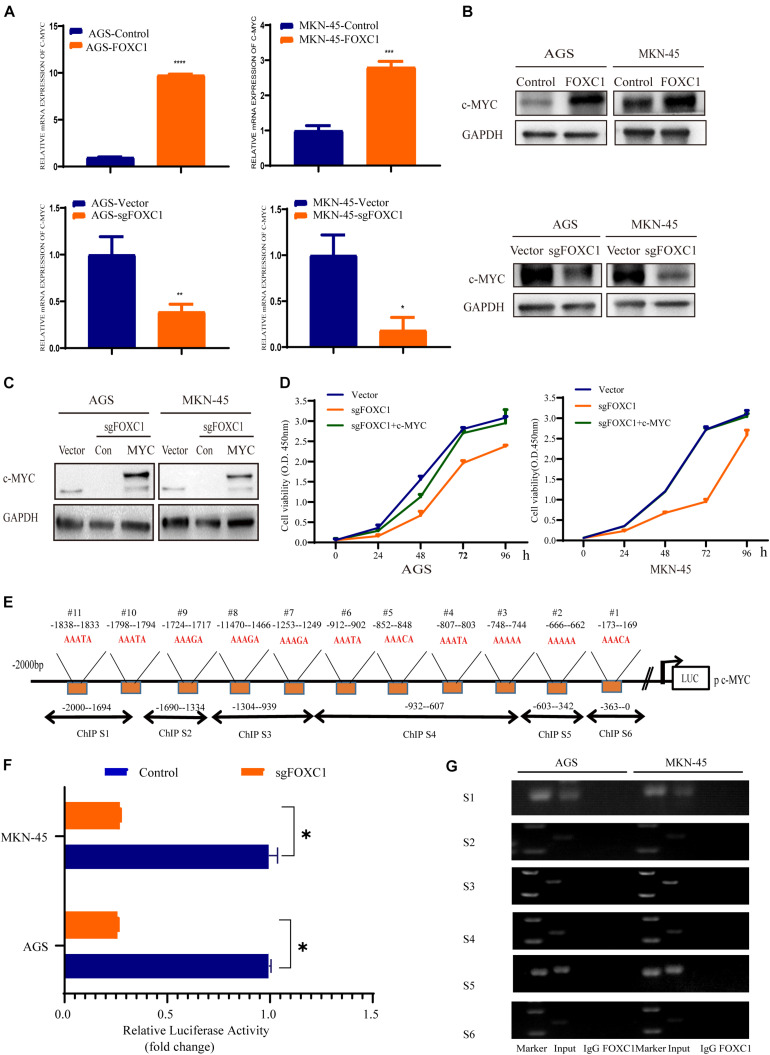
FOXC1 enhances c-MYC expression to regulate gastric cancer cell proliferation. **(A,B)** The results of qPCR **(A)** and western blot **(B)** assays indicated that knockout of FOXC1 in AGS and MKN-45 cells dramatically decreased c-MYC expression but that forced FOXC1 expression in AGS and MKN-45 cells dramatically enhanced c-MYC expression at transcriptional and translational level. Representative figure of the three replicates was shown. **(C)** Validation of ectopic expression of c-MYC in FOXC1 KO AGS and MKN-45 cells using Western blotting. **(D)** Ectopic overexpression c-MYC in FOXC1 KO AGS and MKN-45 cells could restore the FOXC1 deficiency-induced phenotype of cell proliferation. All CCK-8 assays were performed three times and showed the same trend and representative results were shown in panel **(D)**. **(E)** The putative binding site of FOXC1 in the 5′ untranslated regions of c-MYC promoter. **(F)** A double luciferase activity assay in AGS and MKN-45 cells indicated that FOXC1 regulates c-MYC expression at the transcriptional level. **(G)** ChIP assay results showed that there was no potential direct binding site of FOXC1 in c-MYC promoter. *P < 0.05, **P < 0.01, ***P < 0.001, ****P < 0.0001.

Due to c-MYC is a critical transcription factor in regulating cell proliferation and development, we focused on c-Myc in regulating GC proliferation. To investigate the role of c-MYC in FOXC1-mediated GC proliferation, we next analyzed whether c-MYC expression is changed in AGS and MKN-45 cells with KO and overexpression of FOXC1, respectively. The results of qPCR and western blotting indicated that FOXC1 KO in AGS and MKN-45 cells decreased c-MYC expression, and consistently, FOXC1 overexpression dramatically enhanced c-MYC expression (Figures 4A,B). Furthermore, direct overexpression of c-MYC restore the FOXC1-deficient phenotype on cell proliferation in AGS and MKN-45 cells (Figures 4C,D). The results of luciferase activity assays indicated that FOXC1 could regulate c-MYC expression at the transcriptional level (Figures 4E,F). Given that FOXC1 is an important transcription factor, we next examined whether FOXC1 acts as a transcription factor to activate c-Myc expression in AGS and MKN-45 cells. We employed ChIP assay to confirmed whether there was a potential binding site in the c-MYC promoter. However, the results showed no direct binding of FOXC1 to the c-MYC promoter (Figure 4G).

### FOXC1 Expression Enhances c-MYC Expression to Promote Gastric Cancer Cell Proliferation Through Activation of the Wnt Signaling Pathway

**FIGURE 5:**
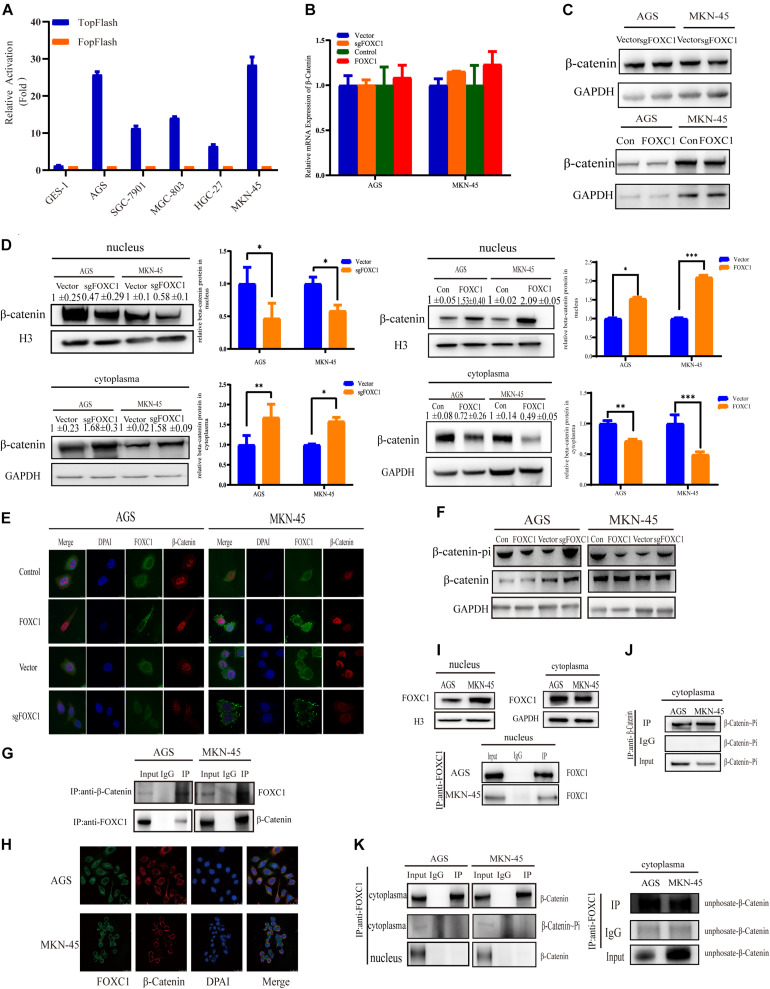
FOXC1 enhances c-MYC expression to promote gastric cancer cell proliferation through activation of the Wnt signaling pathway. **(A)** TopFlash/FopFlash assays using normal gastric cells (GES-1) and gastric cancer cells (AGS, SGC-7901, MGC-803, MKN-45, HGC-27). **(B)** qPCR results in FOXC1 KO and FOXC1-overexpressing AGS and MKN-45 cells indicated that mRNA levels of β-catenin remained unchanged. **(C)** The results of western blot assays in FOXC1 KO and FOXC1-overexpressing AGS and MKN-45 cells indicated that protein levels of β-catenin remained unchanged (β-catenin Antibody: CST #8480S). **(D)** FOXC1 KO increased β-catenin levels in the cytoplasm but decreased them in the nucleus (β-catenin Antibody: CST #8480S). **(E)** Immunofluorescence assay in FOXC1 KO and FOXC1-overexpressing AGS and MKN-45 cells showed that the level of β-catenin translocation into nucleus changed with knockout and overexpression FOXC1 (β-catenin Antibody: CST #8480S). **(F)** FOXC1 overexpression inhibit the phospho-β-Catenin (Ser33/37/Thr41) in AGS and MKN-45 cells (phospho-β-Catenin (Ser33/37/Thr41) Antibody: CST #9561T). **(G,H)** co-IP assays and immunofluorescence assay indicated that FOXC1 binds to the β-catenin protein in the cytoplasm (β-catenin Antibody: CST #8084S; FOXC1 Antibody: Abcam #ab227977). **(I)** Western blot assays and immunoprecipitation assay were applied to confirmed the distribution of FOXC1 in the nucleus. **(J)** co-IP assay was applied to confirmed the distribution of phospho-β-Catenin (Ser33/37/Thr41) in the nucleus (phospho-β-Catenin (Ser33/37/Thr41) Antibody: CST #9561T). **(K)** co-IP assay was applied to confirmed whether FOXC1 bound to the β-catenin protein in the cytoplasm and nucleus. Immunoprecipitation assay results showed that FOXC1 bound to non-phospho-β-Catenin in the cytoplasm (β-catenin Antibody: CST #8084S; phospho-β-Catenin (Ser33/37/Thr41) Antibody: CST #9561T; Non-phospho-β-Catenin (Ser33/37/Thr41) Antibody: CST #8814T), and FOXC1 did not bound to β-Catenin in the nucleus (β-catenin Antibody: CST #8084S). *P < 0.05, **P < 0.01, ***P < 0.001.

Considering c-MYC is an important target gene of the Wnt pathway, we hypothesized that FOXC1 may activate Wnt signaling to enhance c-MYC expression. Given that AGS and MKN-45 cells expressed higher levels of FOXC1 than SGC-7901, MGC-803, HGC-27 cells. Further, Wnt activity was aberrant higher in AGS and MKN-45 cells than in SGC-7901, MGC-803, HGC-27 cells, as determined by TOP-Flash reporter assay (Figure 1A), indicating that Wnt activity is regulated and correlated with the FOXC1 levels in the cells (Figure 5A). β-Catenin aberrant cumulation in the cytoplasm is an important part for Wnt activation. We conducted qPCR and WB assay to explore the potential correlation of FOXC1 and β-Catenin, and the results indicated that FOXC1 did not have a significant influence on β-catenin expression at transcription or translation level (Figures 5B,C). At the same time, we learned that the activity of the wnt signaling pathway is related to β-Catenin in the nucleus (Zhang et al., 2011). Thus, we further explore the distribution of β-catenin in cytoplasm and nucleus. Interestingly, the results showed that β-catenin distribution in the nucleus increased with upregulation of FOXC1, while decreased with FOXC1 knockout, supporting the role of FOXC1 in activation of classic Wnt pathway (Figures 5D,E). In addition, β-Catenin will be labeled with E3 ubiquitin ligase after β-Catenin phosphorylation at Ser33/37/Thr41 in the cytoplasm (Reya and Clevers, 2005; Clevers and Nusse, 2012; Zhan et al., 2017). We also found β-Catenin phosphorylation at Ser33/37/Thr41 increased with FOXC1 KO and decreased with FOXC1 overexpression (Figure 5F), supporting the conclusion that WNT signaling pathway is activated under the condition of high FOXC1 expression. Previous studies indicated FOXM1 could directly bound to β-catenin *in vivo* and *in vitro* and Promoted β-catenin nuclear localization and controls Wnt target-gene expression (Zhang et al., 2011). Considering FOXC1 and FOXM1 belong to forkhead box family, thus we further explore the potential role of FOXC1in nuclear localization of β-catenin. Our results showed that FOXC1bound to β-catenin to form a complex in AGS and MKN-45 cells (Figures 5G,H). However, we also found a contradiction, that is, in Figure 4G, the ChIP experiment showed that there was no potential FOXC1 binding site in c-MYC promoter. Thus, we conducted COIP assays in the cytoplasm and nucleus, and the results showed FOXC1 bound to β-catenin in cytoplasm, but not in nucleus (Figures 5I–K). These results were consistent with previous result demonstrating that FOXC1 does not directly bind to the c-MYC promoter.

### FOXC1 Promotes GC Cell Proliferation via Downregulation of DKK1

**FIGURE 6:**
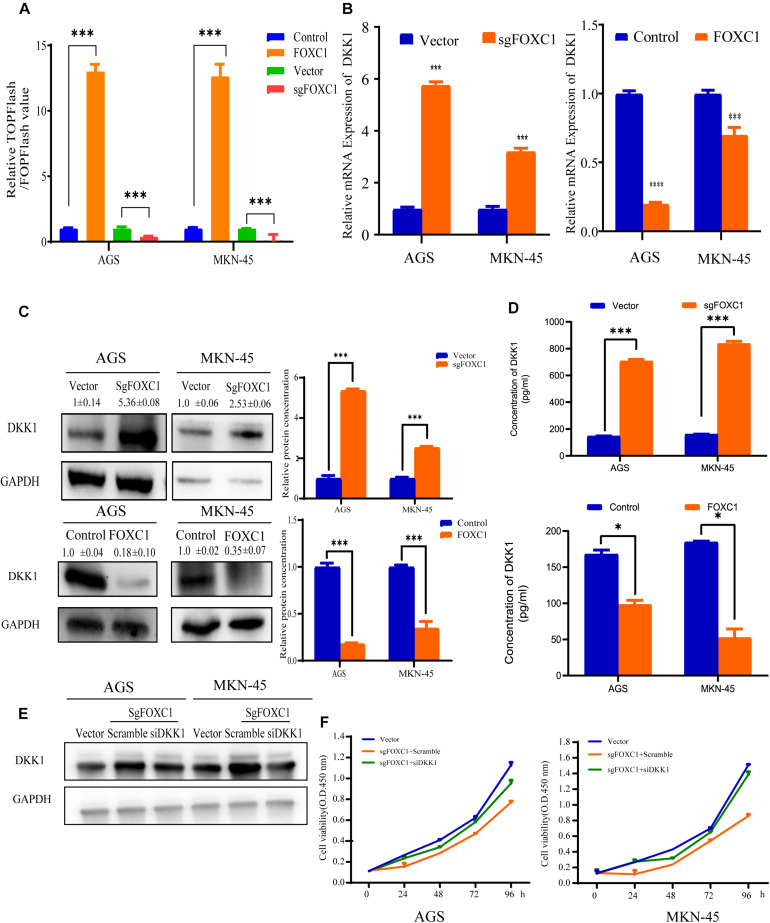
FOXC1 promotes GC cell proliferation via downregulation of DKK1. **(A)** TopFlash/FopFlash assays were used to investigate activity of the Wnt signaling pathway in FOXC1 KO and FOXC1 overexpression AGS and MKN-45 cells. The results demonstrated that FOXC1 KO decreased activity of the Wnt signaling pathway and forced FOXC1 expression could increase activity of the Wnt signaling pathway. **(B–D)** qPCR **(B)**, western blot **(C)** and ELISA **(D)** assays in FOXC1 knockout GC cells were used to analyze the mRNA and protein levels of DKK1. **(E)** Western blot assay was applied to confirmed the knockdown efficiency of DKK1 in FOXC1 KO AGS and MKN-45 cells. **(F)** DKK1 knockdown could restore FOXC1 KO-induced suppression of AGS and MKN-45 cell proliferation. All CCK-8 assays were performed three times and showed the same trend and representative results were shown inpanel **(F)**. *P < 0.05, ***P < 0.001.

To find out the mechanism by which FOXC1 activates the Wnt signaling pathway, we first applied the TopFlash/FopFlash assay to determine the activity of Wnt pathway in FOXC1 KO GC cells. The results indicated that the activity of the Wnt signaling pathway was decreased in FOXC1 KO and increased in FOXC1 overexpression AGS and MKN-45 cells (Figure 6A). Considering the role of DKK1 in classic Wnt signaling, We next examined the level of DKK1 expression, which is the antagonist of Wnt signaling, and found that the DKK1 mRNA level increased in FOXC1 KO GC cells and decreased in FOXC1-overexpressing GC cells (Figure 6B; Zhu et al., 2021). At the same time, we applied western blot and EILSA assays were to confirm the correlation of DKK1 protein and FOXC1, and results indicated that FOXC1 negatively regulated DKK1 expression at translational level (Figures 6C,D). In addition, siDKK1 restored the cell proliferation repressed by FOXC1 KO in AGS and MKN-45 cells (Figures 6E,F). Those results indicated FOXC1 regulated GC cells proliferation through DKK1.

### DKK1 Is a Downstream Target of FOXC1 in GC

**FIGURE 7:**
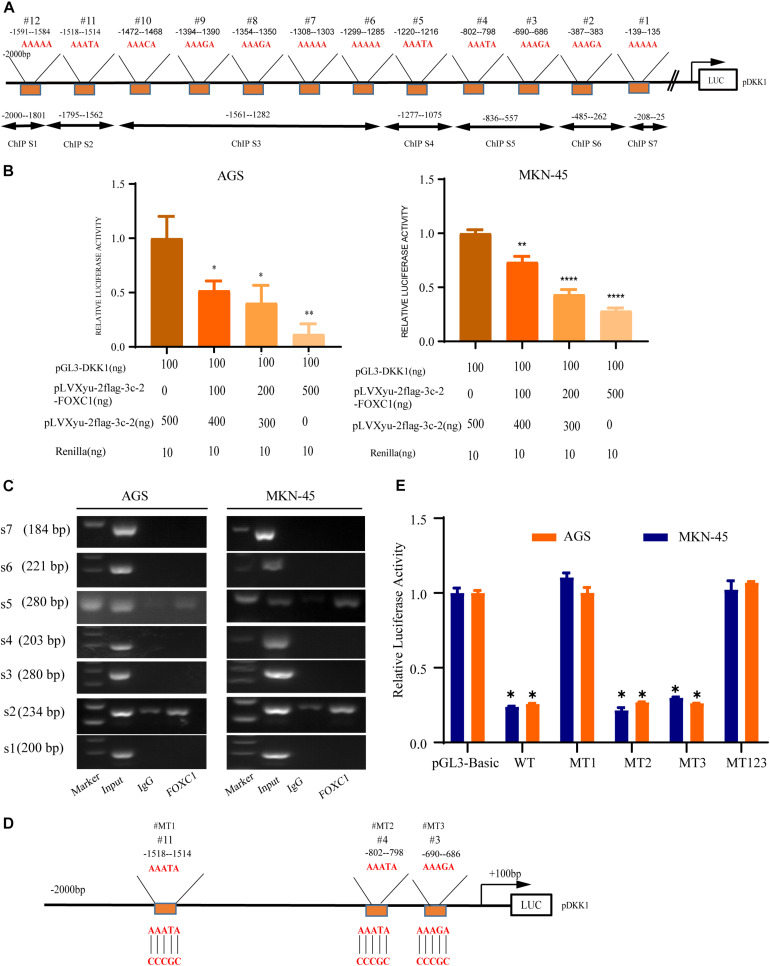
DKK1 is downstream of FOXC1 in GC. **(A)** Considering the specific DNA sequence for FOX transcription factor (AAAYA), A total of 12 putative FOXC1 binding sites in the 5’ untranslated region of DKK1promoter. **(B)** Double luciferase activity assay in AGS and MKN-45 cells to confirm the influence of FOXC1 on the transcription of DKK1. The results of Luciferase activity were dose-dependently decreased after co-transfection with FOXC1 (*P < 0.05), which is also indicated that FOXC1 negatively regulated DKK1 expression at transcriptional level. **(C)** Chromatin immunoprecipitation assay (ChIP) results demonstrated that FOXC1 binds directly to the DKK1 promoter at ChIP S2 and ChIP S5 in the **(A)**, which were consisted by three putative binding sites (site3#, site4# and site 11#). **(D,E)** Schematic diagram of mutation strategies in the DKK1 promoter. The results indicated that FOXC1 decreased the luciferase activity of the WT DKK1 promoter to approximately 75% of the corresponding control but did not affect the activity of MT123 or MT1 (*P < 0.05, **P > 0.05, ****P < 0.0001).

A previous study indicated that AAAYA-rich sequences are the main DNA-binding sequence of FOX transcription factors (Berry et al., 2002), and we found 12 potential binding sites (AAAYAs) in the DKK1 promoter (Figure 7A). To evaluate possible interaction between DKK1 and FOXC1, the sequence from + 100bp to −2000bp of the human DKK1 promoter was cloned into a luciferase reporter plasmid (pGL3-DKK1-Luc), and pGL3-DKK1-Luc plasmids with FOXC1 plasmids or control vectors were transiently transfected into AGS and MKN-45 cells. Luciferase assays indicated that FOXC1 significantly decreased the luciferase activity of DKK1 in both cell lines (Figure 7B). According to ChIP analysis, FOXC1 directly binds to the DKK1 promoter (sites #2 and #5) (Figure 7C). To further validate whether DKK1 is regulated directly by FOXC1, we used the wildtype (WT) or mutant (MT) promoter of DKK1 in the luciferase assay (Figure 7D). We found that FOXC1 reduced the luciferase activity of the WT DKK1 promoter to approximately 75% of the corresponding control but did not affect the activity of MT1 or MT123, suggesting that FOXC1 binds to the site #1 (–1514–1518 bp) in the DKK1 promoter (Figure 7E).

## Discussion

Wnt signaling pathway is highly activated in gastric cancer (GC) and can contribute growth and metastasis of GC (Zhao et al., 2014). Many studies explored the function of Wnt signaling pathway in tumor progression, however, the reason why it is highly activated in GC remains to be studied. Research to date on this pathway has mostly focused on positive regulatory factors, whereas its antagonist proteins have attracted little attention (Santos et al., 2016; Gao et al., 2018; Nanki et al., 2018; Yang et al., 2018). In this study, we found that FOXC1 was elevated in GC tissue and its level was associated with patients, poor prognosis. Furthermore, the results showed that FOXC1 can promote growth of GC by modulating the tumor cell cycle, as mediated by c-MYC and cyclin D1, further supporting the role of FOXC1 in GC progression (Wang et al., 2018, 2020).

c-Myc is a critical transcription factor, and its aberrant expression of c-Myc can promote tumorigenesis and development (Sansom et al., 2007). For many years, c-Myc has been considered as a potential drug target for suppressing tumors, however, drug development for the c-Myc target itself has always been a difficult point due to the absence of a suitable pocket for high-affinity binding by low molecular weight inhibitors (Duffy et al., 2021). Recent studies have also shown that c-Myc is an important target gene of the wnt signaling pathway, and pharmacologic targeting of Fzd could effectively inhibited the growth of gastric adenomas by influence the expression of c-Myc (Flanagan et al., 2019; Ashrafizadeh et al., 2020). Therefore, inhibiting the activity of the Wnt pathway can significantly reduce the expression of c-Myc and inhibit the proliferation of tumor cells. In this study, we focused on c-Myc in regulating GC proliferation. While restore c-Myc expression could reverse the inhibition effect of FOXC1 knockout. And the results in this study indicated that, c-Myc is positively correlated with FOXC1 expression. Due to FOXC1 is a transcription factor, we explore whether c-Myc is a direct target gene of FOXC1. However, the result of ChIP assay showed that there were no potential binding sites in c-Myc promoter. Thus, we turned to focus on the activity of Wnt pathway in GC cells. Interestingly, the activity of Wnt pathway increased with FOXC1 overexpression and increased with FOXC1 knockout. Besides, the transcription of c-Myc also changes with Wnt signal pathway, which is consistent with the results of previous studies (Ramezani et al., 2019).

In further research, we interfere the expression of FOXC1 in AGS and MKN-45 cells. Expression of DKK1 was changed with knockout and overexpression of FOXC1 at transcriptional and translational level. These results indicate that FOXC1 might negatively regulate expression of DKK1. Considering the role of DKK1 in classic Wnt signaling, we sought to clarify the function of FOXC1 in GC proliferation by regulating DKK1 expression (Zhu et al., 2021). Our study indicated that DKK1 expression negatively correlated with proliferative ability in GC cells, which is consistent with the results of a previous study of DKK1 in GC proliferation (Wang et al., 2013; Hong et al., 2018). These results suggested that FOXC1 regulated GC cell proliferation by regulating the expression of DKK1. Considering the role of FOXC1 and DKK1 expression in GC cells proliferation, we want to classify the specific mechanisms of FOXC1 and DKK1 expression in GC cells. Due to FOXC1 is a transcription factor, we further investigated whether DKK1 is a direct transcription target of FOXC1. Through a dual luciferase reporter assay and ChIP assay, we found that FOXC1 act as a transcription factor and directly bound to the DKK1 promoter region (-1518–1514) to negatively regulate transcription of this gene. Although there were three direct binding sites in the result of ChIP assay, the result of luciferase verified that there was only one site that were confirmed had a significant influence on DKK1 transcription due to the help of specific transcription cofactors, which is crucial to perform transcription activation functions. As for which transcriptional cofactors are, it remains to be further explored. At the same time, knockdown DKK1 expression in FOXC1 knockout cells could restore the effect of FOXC1 in promoting proliferation, which indicated the importance of FOXC1/DKK1 axis in GC proliferation.

**FIGURE 8:**
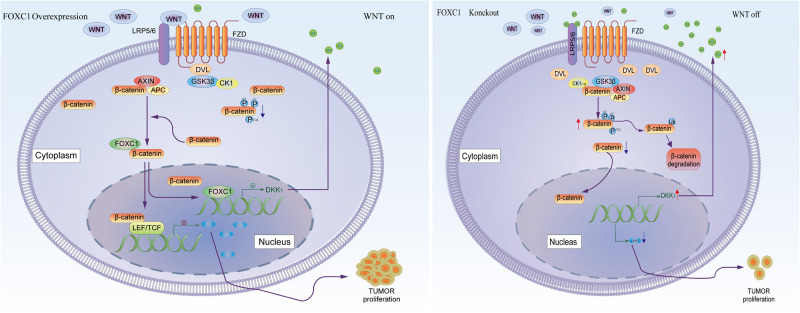
Schematic depicting the role of FOXC1 and DKK1 in the regulation of gastric cancer proliferation. FOXC1 is overexpressed in gastric cancer cells and acts as a transcription factor that negatively regulates DKK1 expression by binding to its promoter region, thereby modulating the activation state of the Wnt signaling pathway and upregulating expression of c-MYC, promoting the proliferation of GC cells. At the same time, FOXC1 also forms a complex with the unphosphorylated β-catenin (Ser33/37/Thr41) protein in the cytoplasm but dissociates in the nucleus.

β-Catenin aberrant cumulation in the cytoplasm is an important part for Wnt activation. In gastric cancer cells, the level expression of FOXC1 is associated with aberrant activation of Wnt pathway due to FOXC1 binding to DKK1 promoter to negatively regulated its transcription, which could affect the level of phosphate-β-Catenin at Ser33/37/Thr41 in the cytoplasm and contributed β-catenin aberrant cumulation in the cytoplasm. It is reported that FOXM1 could directly bound to β-catenin and Promoted β-catenin nuclear localization and controls Wnt target-gene expression (Zhang et al., 2011). Considering FOXC1 and FOXM1 belong to forkhead box family, thus we further explore the potential role of FOXC1in nuclear localization of β-catenin. Our results showed that FOXC1 did not have a significant influence on β-Catenin at transcriptional level or translational level, it affected the level of phosphate-β-Catenin at Ser33/37/Thr41 in the cytoplasm and nuclear entrance process of β-Catenin. In addition, FOXC1bound to unphosphorylated β-catenin to form a complex in the cytoplasm, and FOXC1 did not bind to β-catenin after the complex enters the nucleus (Figure 8). However, we did not clarify the specific mechanism of FOXC1 promotes β-catenin translocation into the nucleus. Therefore, we will further study the underlying mechanism by which FOXC1 regulates the function of β-catenin in future research.

In conclusion, our study revealed the function of FOXC1/DKK1 in GC cell proliferation, which provided a potential biomarker and target therapy for GC.

## Data Availability Statement

Publicly available datasets were analyzed in this study. This data can be found here: http://gepia.cancer-pku.cn/detail.php?gene=FOXC1; http://kmplot.com/analysis/index.php?p=service&cancer=gastric.

## Ethics Statement

The studies involving human participants were reviewed and approved by The human sample study was reviewed and approved by Shanghai Jiao Tong University School of Medicine. The patients/participants provided their written informed consent to participate in this study. The animal study was reviewed and approved by the animal study was reviewed and approved by Shanghai Jiao Tong University School of Medicine.

## Author Contributions

HZ, JD, and FY conceived and designed the studies. JJ and HZ conducted most of the experiments and analyzed the data. JJ, BS, and WW conducted all animal studies. FY performed immunohistochemical technology and data analysis. JL provided technical support for some cell experiments. JJ and WY wrote this manuscript. All authors contributed to the article and approved the submitted version.

## Conflict of Interest

The authors declare that the research was conducted in the absence of any commercial or financial relationships that could be construed as a potential conflict of interest.

## Footnotes

1 http://kmplot.com/analysis/index.php?p=service
