# Supplementary material for: FOXC1 Negatively Regulates DKK1 Expression to Promote Gastric Cancer Cell Proliferation Through Activation of Wnt Signaling Pathway
Source: Front Cell Dev Biol. 2021 Apr 27;9:662624. doi: 10.3389/fcell.2021.662624 (PMC8111291; doi:10.3389/fcell.2021.662624)
Supplement: Supplementary file 1 [file Table_1.DOCX]

Quantitative real time polymerase chain reaction assay (Q-PCR) primer

| Gene name | Sequence detail information |
| --- | --- |
| GAPDH-Forward | GCACCGTCAAGGCTGAGAAC |
| GAPDH-Reverse | TGGTGAAGACGCCAGTGGA |
| FOXC1 -Forward | AACAGCATCCGCCACAACCTC |
| FOXC1-Reverse | TCCTTCTCCTCCTTGTCCTTCAC |
| YAP-Forward | TGTCCCAGATGA ACGTCACAGC |
| YAP-Reverse | TGGTGGCTGTTT CACTGGAGCA |
| TAZ -Forward | CACCGTGTCCAATCACCAGTC |
| TAZ -Reverse | TCCAACGCATCAACTTCAGGT |
| β-catenin -Forward | CATCTACACAGTTTGATGCTGCT |
| β-catenin - Reverse | GCAGTTTTGTCAGTTCAGGGA |
| AXIN1 -Forward | GGTTTCCCCTTGGACCTCG |
| AXIN1 -Reverse | CCGTCGAAGTCTCACCTTTAATG |
| NOTCH1 -Forward | GAGGCGTGGCAGACTATGC |
| NOTCH1 -Reverse | CTTGTACTCCGTCAGCGTGA |
| BIRC5 -Forward | AGGACCACCGCATCTCTACAT |
| BIRC5 -Reverse | AAGTCTGGCTCGTTCTCAGTG |
| CCND1-Forward | GCTGCGAAGTGGAAACCATC |
| CCND1-Reverse | CCTCCTTCTGCACACATTTGAA |
| c-MYC -Forward | GTCAAGAGGCGAACACACAAC |
| c-MYC -Reverse | TTGGACGGACAGGATGTATGC |
| SPP1 -Forward | GAAGTTTCGCAGACCTGACAT |
| SPP1 -Reverse | GTATGCACCATTCAACTCCTCG |
| GPC3 -Forward | ATTGGCAAGTTATGTGCCCAT |
| GPC3 -Reverse | TTCGGCTGGATAAGGTTTCTTC |
| BAX -Forward | CCCGAGAGGTCTTTTTCCGAG |
| BAX -Reverse | CCAGCCCATGATGGTTCTGAT |
| PUMA-Forward | GCCAGATTTGTGAGACAAGAGG |
| PUMA-Reverse | CAGGCACCTAATTGGGCTC |
| DKK1 -Forward | CTCGGTTCTCAATTCCAACG |
| DKK1 -Reverse | GCACTCCTCGTCCTCTG |
