# Supplementary material for: FOXC1 Negatively Regulates DKK1 Expression to Promote Gastric Cancer Cell Proliferation Through Activation of Wnt Signaling Pathway
Source: Front Cell Dev Biol. 2021 Apr 27;9:662624. doi: 10.3389/fcell.2021.662624 (PMC8111291; doi:10.3389/fcell.2021.662624)
Supplement: Supplementary file 2 [file Table_2.DOCX]

Supplementary materials (the detail information of antibodies)

| Name | Brand | Catalog number |
| --- | --- | --- |
| GAPDH Monoclonal antibody | proteintech | 60004-1-Ig |
| FOXC1(WB/IHC/ ICC/IF/IP) | Abcam | **ab227977** |
| YAP1 | HUABIO | ET1608-30 |
| TAZ | HUABIO | ER1917-5 |
| β-catenin | CST | 8480S |
| Non-phospho-β-Catenin (Ser33/37/Thr41) | CST | 8814T |
| Phospho-β-Catenin (Ser33/37/Thr41) Antibody | CST | 9561T |
| CCND1 | HUABIO | ET1601-3 |
| Notch1 | HUABIO | ET1606-55 |
| c-MYC | Abcam | ab32072 |
| DKK1 | HUABIO | ET1610-63 |
| AXIN1 | HUABIO | ER6275 |
| H3 | CST | 4499S |
| FOXC1(CHIP) | Abcam | ab5079 |
